# Medical physics staffing for radiation oncology: a decade of experience in Ontario, Canada

**DOI:** 10.1120/jacmp.v13i1.3704

**Published:** 2012-01-05

**Authors:** Jerry J. Battista, Brenda G. Clark, Michael S. Patterson, Luc Beaulieu, Michael B. Sharpe, L. John Schreiner, Miller S. MacPherson, Jacob Van Dyk

**Affiliations:** ^1^ Medical Physics London Regional Cancer Program London ON; ^2^ Radiation Medicine Program The Ottawa Hospital Cancer Centre Ottawa ON; ^3^ Juravinski Cancer Centre and McMaster University Hamilton ON; ^4^ Université Laval Québec QC; ^5^ Radiation Medicine Program Princess Margaret Hospital and University of Toronto Toronto ON; ^6^ Cancer Centre of South Eastern Ontario Kingston ON Canada

**Keywords:** staffing, medical physics, radiation oncology

## Abstract

The January 2010 articles in *The New York Times* generated intense focus on patient safety in radiation treatment, with physics staffing identified frequently as a critical factor for consistent quality assurance. The purpose of this work is to review our experience with medical physics staffing, and to propose a transparent and flexible staffing algorithm for general use. Guided by documented times required per routine procedure, we have developed a robust algorithm to estimate physics staffing needs according to center‐specific workload for medical physicists and associated support staff, in a manner we believe is adaptable to an evolving radiotherapy practice. We calculate requirements for each staffing type based on caseload, equipment inventory, quality assurance, educational programs, and administration. Average per‐case staffing ratios were also determined for larger‐scale human resource planning and used to model staffing needs for Ontario, Canada over the next 10 years. The workload specific algorithm was tested through a survey of Canadian cancer centers. For center‐specific human resource planning, we propose a grid of coefficients addressing specific workload factors for each staff group. For larger scale forecasting of human resource requirements, values of 260, 700, 300, 600, 1200, and 2000 treated cases per full‐time equivalent (FTE) were determined for medical physicists, physics assistants, dosimetrists, electronics technologists, mechanical technologists, and information technology specialists, respectively.

PACS numbers: 87.55.N‐, 87.55.Qr

## I. INTRODUCTION

Medical physicists provide essential technical and scientific support to radiation oncology practices through a combination of direct support of patient treatment, routine quality control of treatment‐related technology, and the development and improvement of treatment procedures. Unlike many other medical professionals for whom the staffing may be directly related to such parameters as the number of patients seen or treated, the diversity of workload and the continually changing technology have rendered the assessment of physics staffing a challenging problem that demands a balanced approach accounting for factors such as technological environment, regulatory requirements, quality and safety, and the number of patients treated.^(^ ^1^ ^–^ ^7^ ^)^

The need for appropriate physics staffing has been reinforced in the analysis of adverse incidents where inadequate physics staffing has been implicated,^(^ ^8^ ^–^ ^17^ ^)^ most recently in the widely discussed articles in *The New York Times*,^(^ ^18^ ^)^ and of particular relevance to Ontario, the 2007 underdosing of 326 patients treated at The Ottawa Hospital Cancer Centre.^(^ ^11^ ^)^ Appropriate staffing standards for radiation oncology must account for the needs of the local patient populations, the capacity to provide radiation therapy in a timely fashion, and the technological demands of new treatments. Historically, various approaches to staffing strategies have been explored, incorporating such factors as the volume of radiotherapy cases, time to perform specified tasks, and the demands of maintaining the required technical infrastructure. A review of recent U.S., European, UK, and Asia‐Oceanic documents demonstrates the increasing importance of accounting for factors beyond a simple patient census.^(^ ^4^ ^,^ ^6^ ^,^ ^19^–^24^ ^)^ All of these jurisdictions recognize a rapid and pervasive technological change that continues to affect radiation therapy practice. Additional physics staffing is needed to maintain treatment and imaging equipment, to provide additional patient‐specific QA for high‐precision treatment techniques, to support information technology for the exchange of complex planning and treatment information, and to balance workload via supervised delegation of clinical service responsibilities. The need to enhance training programs and to provide continuing education and professional development within medical physics departments and to other allied radiation therapy specialties is also discussed by these organizations. Training programs must align with demands associated with general population growth, an aging population, geographic redistribution, radiotherapy utilization levels, broad health policy objectives to improve access to care, and shifting employment demographics relating to changing retirement policies, parental leave, and job‐sharing arrangements.^(^ ^7^ ^,^ ^25^–^26^ ^)^ Furthermore, radiation safety regulatory requirements, both for radioactive isotope management and therapy treatment unit licensing, have become more complex and require additional administration.

In the United States, professional societies have analyzed the workload associated with providing professional reimbursed physics services.^(^ ^3^–^5^ ^,^ ^26^–^27^ ^)^ Through the well‐known series of studies published by Abt consultants,^(^ ^3^ ^–^ ^5^ ^)^ relative work units have been determined by capturing estimates of the time expended in various procedures defined by specific billing codes. These studies are widely recognized by administrative professionals as justification for basic staffing needs of medical physics. While often cited as de facto standards, the Abt studies are also criticized for not addressing an appropriate mix of personnel, academic activity or the supervision of the technical work of support staff specialized in physics, medical dosimetry, engineering, and information technology. Additionally, these guidelines have been revised at regular intervals to account for changes in technology.^(^ ^26^ ^)^ Mills et al.^(^ ^19^ ^)^ have discussed U.S. trends in supply and demand for radiation oncology physics services, in particular with reference to the upcoming changes in eligibility requirements for U.S. certification of radiation oncology physicists to be implemented by the American Board of Radiology (ABR) in the years 2012/2014. They concluded that there is a clear and urgent need to expand the capacity of graduate and residency training programs for medical physics well in advance of the certification examination deadlines.

Recently, the American Association of Physicists in Medicine (AAPM) commissioned a comprehensive workforce study that reviewed current staffing and developed simulation models of supply and demand to understand the potential workforce ramifications of the changing education requirements for ABR certification.^(^ ^27^ ^)^

Klein^(^ ^20^ ^)^ has described a detailed staffing grid methodology to estimate the full‐time equivalent (FTE) requirements to meet the full spectrum of medical physics activities including administrative, regulatory, educational, developmental, and technical demands. The most recent staffing guidelines from the United Kingdom's Institute of Physics and Engineering in Medicine (IPEM)^(^ ^6^ ^)^ were also based on a grid model that accounts for the number and complexity of treatment techniques, equipment inventory, and department‐wide factors related to administration, radiation protection, and quality assurance. The model estimates the number of whole‐time equivalent (WTE) staff required by a department, an approach very similar to the original approach adopted for Ontario in 1989.^(^ ^28^ ^)^

ESTRO^(^ ^21^ ^–^ ^22^ ^)^ has conducted a survey of guidelines for radiotherapy infrastructure and staffing requirements from 41 countries across Europe and observed a large variation depending on local equipment and practices. Its most recent guideline is to hire one physicist per linear accelerator treating 450 to 500 patients per year, noting that this estimate does not address the most modern of radiotherapy practices and the diversity of radiotherapy techniques, nor does is take into account nonclinical activities such as teaching or professional development. Holmberg and McClean^(^ ^29^ ^)^ examined how complexity influences treatment planning workload, and developed a method for predicting the resultant effect on staffing requirements. The model classifies treatment plans according to their complexity, based on a retrospective analysis of the time required to complete planning tasks of various complexity on similar patients. The authors suggest their model should be populated with a local dataset based on time studies and validated prior to adoption.

In Canada, the Canadian Partnership Against Cancer (CPAC) is a federal cancer strategy initiative connecting federal health agencies, provincial cancer programs, associations representing health care workers, patient advocacy groups, and others. CPAC's Human Resources Working Group^(^ ^7^ ^)^ has issued a comprehensive report highlighting the need to plan medical physics staffing needs to assure current staffing meets the requirements for appropriate patient care and technical development, to establish training needs, and to set baselines to determine future human resource requirements.

The purpose of this report is to share our experience of the impact of new technology on staffing levels over the past decade in the province of Ontario, Canada, and to propose a revised algorithm to estimate staffing levels as a function of local radiotherapy practice and infrastructure. The generality of the methodology was tested through a survey of cancer centers across Canada and with a comparison to current and ideal staffing used as a guide to assess appropriateness of our calculations. In addition, simplified planning guidelines (annual treated cases per FTE) are estimated for large‐scale planning of human resources at the provincial or national level.

### Background to Ontario medical physics staffing

#### Radiotherapy in the Province of Ontario

The province of Ontario spans more than $1,000,000\;{\text{km}}^{2}$ and accounts for one‐third of the Canadian population (13,000,000 residents).^(^ ^30^ ^)^ Health care services in Canada are administered provincially, based on national guidelines within a social funding framework. Access to radiation therapy is generally coordinated by a provincial agency responsible for planning the allocation of cancer treatment infrastructure, providing leadership for continual improvement of cancer services through practice guidelines, and setting service and research priorities. In Ontario, this agency is Cancer Care Ontario (CCO).^(^ ^31^ ^)^ Ontario is now served by 12 regional cancer centers (with a further two under construction) integrated with local host hospitals throughout the province, eight of which are integrated with a university medical school.

From 2000 to 2009, Ontario's population rose from 11.4 million to 13 million and the annual radiotherapy caseload increased from approximately 24,000 to 34,000, of which approximately 20% are retreatments and 3% are non‐melanoma skin cancer treatments. During the same period, for modeling purposes, the cancer incidence was assumed to rise by 3% per annum due to population growth and ageing. In reality, the radiotherapy caseload increased by an average of 3.7% per annum, reflecting improved access and utilization of radiation.^(^ ^32^ ^–^ ^33^ ^)^ The number of radiation facilities has grown from nine to 14, and the number of accelerators increased from 63 to 86 over the decade, with an additional 20 machines anticipated to meet demand and increase capacity within the next 5–10 years. The current rate of utilization of radiation for the treatment of primary malignant disease (excluding non‐melanoma skin cancer) in Ontario is 35.5%. The goal is to increase this to 48% over a period of 10 years, to approach the rate of 52% recommended by Delaney et al.^(^ ^34^ ^)^

**Figure 1:**
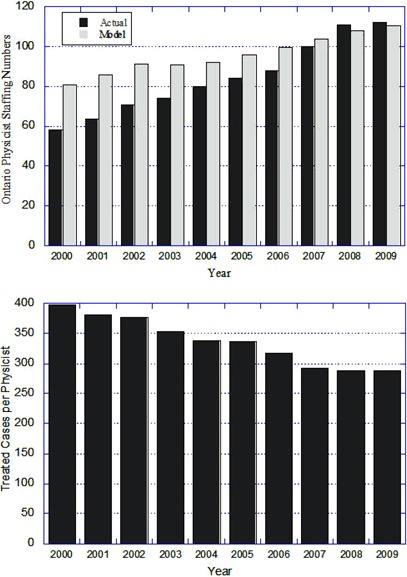
Top: actual total number of medical physicists (FTE) in Ontario cancer centers for the period 2000–2009 (dark grey) compared to the provincial guideline of “1 physicist per 300 treated cases” (light grey); Bottom: ratio of annual treated cases per physicist for the same period.

Figure 1 shows the Ontario staffing for medical physicists over the past decade. The number of medical physicists in radiation oncology has risen from 58 to 123 FTE (as of November 2010). This growth rate in staffing is similar to that experienced in Australasia (7% per annum between 2006 to 2009^(^ ^35^ ^–^ ^36^ ^)^) — although this was during a period when the Australasian governments were actively financially supporting the recruitment and training of new physicists, so growth rate had been accelerated over that period. The Ontario workforce also experienced growth, hiring 64 physicists from a pool of 80 who graduated from the Ontario Physics Residency Program^(^ ^37^ ^)^ and 43 physicists from out‐of‐province. This external recruitment essentially compensated for a loss of 42 physicists through departures, resignations, or retirements, while the residency program fuelled the growth. A more formal medical physics residency program was established through centralized Ontario government funding (at a cost of approximately CND$1,000,000 per year), supporting tuition and competitive stipends for 20 trainees per annum (i.e., 10 for each of two years of training). Funding of a standardized training program has proven to be critical to stabilizing the workforce during the past decade.

Staff projections may be distilled into a rule‐of‐thumb based purely on patient caseload, more easily referenced by hospital administrators and government agencies and more amenable to comparison with other published standards and guidelines. In 1991, for example, the U.S. Inter‐Society Council for Radiation Oncology developed a comprehensive list of radiotherapy procedures published in its “Blue Book”^(^ ^1^ ^)^ and recommended that a minimum of 1 FTE physicist should be on staff per 400 radiotherapy cases. This simpler method is useful for large‐scale applications or intercomparison of staffing across jurisdictions. Podgorsak^(^ ^38^ ^)^ used this method to compare workloads across Canada in the pre‐IMRT era; the figure was then approximately 430 treated cases per physicist for Canada as a whole, but reached approximately 500 in the province of Quebec. Clearly, these data serve as guidance for government decision‐makers.

Staffing needs may also be forecast using cancer incidence and radiotherapy utilization rates available from government organizations. During the past decade, for example, Cancer Care Ontario adopted the staffing ratio of 300 annual treated cases per FTE physicist, derived by averaging the results from the detailed Ontario algorithm. This figure was subsequently also adopted fairly rapidly throughout most jurisdictions in Canada. Referring to Fig. 1, this level of 300 cases per year per FTE was achieved in Ontario in 2007–08, but has since proved inadequate due to the need to support resource‐intensive technologies. In summary, the advantages of this method are simplicity and the obvious link to a measurable end product — a treated radiotherapy case. The disadvantage is that the method relies on a ratio averaged over a wide mix of caseload and clinical practice, without explicit consideration for local variables that affect workload. FTE ratios must also be reviewed periodically to account for changes in clinical treatment options or workload redistribution. For example, in view of the recent evolution of radiotherapy and extensive implementation of IMRT, a comprehensive staffing review is being undertaken by Cancer Care Ontario^(^ ^39^ ^)^ and this current work was prompted in part to provide data for that Ontario review.

## II. METHODS

### Definition of *workforce*

Medical physics services provided in support of radiation oncology in Ontario are delivered through a group of professionals (medical physicists, physics assistants, medical dosimetrists, engineering technologists, and information technology specialists). To facilitate the interpretation of our findings, a brief summary of typical qualifications and duties is provided in Appendix A. The number of medical physicists required must be interpreted in the context of having a complement of such support staff working as a team. In other jurisdictions, a different mix of personnel is often observed and the number of medical physicists must be adjusted accordingly.

### Definition of *caseload*

A precise definition of caseload is also required to understand the algorithm and to permit a valid intercomparison across jurisdictions. As noted previously, the concept of “radiation‐treated cases per year” is used in Ontario. This is an annual count of external beam and brachytherapy cases started on treatment during a given year. New cases as well as retreated cases are counted, with retreatments accounting for approximately 20% of the total. Cases that are started late in a previous year are not counted even though the workload extends into the sampling year. Conversely, treatments that start late in a sampling year are counted and under equilibrium conditions, these two effects should be balanced. Note, however, that multiple “phases” or “episodes” aimed at the same disease site do not increase the count if they are started within the same year. Metastasis is also considered as a single disease entity, even if progression occurs at several anatomical sites (e.g., brain and lung); treatments associated with a single diagnosis are only counted once if executed within the same year. The treated case metric has no “intensity factor” applied for complexity of treatment planning or level of patient‐specific quality assurance required. Despite these limitations in measuring actual workload, this statistic has nonetheless provided a consistent historical reference across the decade of Ontario experience reported here.

### Definition of *full‐time equivalent (FTE)*

In our context, a full‐time equivalent (FTE) corresponds to the work time paid to a full‐time employee for one year (52 weeks) in Ontario. The standard five‐day work week consists of 37.5 paid hours per week. An FTE is, therefore, equivalent to an annual total of 1950 paid hours. This total time includes paid vacation and holidays (typically 0.1 FTE or 26 days per year), and attendance at conferences or courses (typically 2 weeks per year). In the following algorithmic method, a correction is applied to account for the FTE loss due to time away from the workplace. Note that the FTE definition is not reflective of extra hours applied to meet clinical services deadlines or academic expectations, usually worked without explicit compensation.

### A. Method 1: Staffing algorithm

The first Ontario staffing algorithm was developed in early 1989 and included explicit accounting for patient‐related services and for radiotherapy‐related equipment support specific to each cancer center. This method calculates the minimum number of medical physicists, physics assistants, medical dosimetrists, engineering, and information technology technologists required to support each cancer center's workload. FTE coefficients reflected the fraction of an employee's annual paid time that should be committed to completing a specified number of particular tasks per year. For example, the FTE weight for a medical physicist's role in patient‐related services was originally set to 1.0 FTE per 1,000 annual radiotherapy cases per year. Incremental FTE weights were also assigned for equipment commissioning, calibration and support, development of new treatment techniques, teaching of students and residents, and for overall department administration. The advantage of this approach is that the FTE factors may be adjusted periodically to reflect changes in practice and workload redistribution among the various professionals in the radiation oncology program of a particular cancer center. In 1993, the FTE weight for medical physicists was reduced to 0.8 FTE per 1,000 annual cases, reflecting a growing role for dosimetrists in computerized treatment planning. Results from several algorithms were compared for application across Canada, as summarized by Dunscombe.^(^ ^2^ ^)^ With minor modifications, this Ontario algorithm was adopted by the Canadian Organization of Medical Physicists (COMP) in 1995.

In performing a specific task (T), the fractional FTE demand for personnel of category (P) completing N procedures is given by:

(1) $$\text{FTE}(P,N,T)=\frac{N\;\text{procedures}\;\text{of}\;\text{type}\;T\;\text{per}\;\text{year}\times \text{time}\;\text{required}\;\text{per}\;\text{procedure}}{\text{Paid}\;\text{work}\;\text{time}\;\text{per}\;\text{year}}$$

The total FTE demand is then given by the sum of the FTE demands for the entire set of tasks of type T performed to service the annual clinical workload. This approach requires a detailed menu of clearly‐defined services (such as those linked to US billing codes^(^ ^20^ ^)^) and a survey of the time expended on each activity during a sampling period. Table 1 gives a summary of several time‐logging studies completed for medical physics activities.^(^ ^4^ ^,^ ^6^ ^,^ ^20^ ^,^ ^23^ ^,^ ^40^ ^)^ To facilitate presentation of these numbers on a single table, some of the values have been simplified and condensed.

**Table 1:** Times per procedure for four jurisdictions (light grey) converted to FTE weights for Ontario (white). Data from Australasia^(^ ^24^ ^)^ and the UK^(^ ^25^ ^)^ are shown for comparison.

*Times per Procedure*	*Quebec (Beaulieu)*	*WashU (Klein)*	*US (Abt III)*	*Ontario Time (hr)*	*Ontario FTE Weight*	*Asia‐Oceania 2010*	*UK IPEM 2009*
Treatment Planning Procedures (hr/case)							
Baseline Conventional RT case	4.94	2.00	0.78	1.00	0.5/1,000 cases	0.2–1	0.80
IMRT (Inverse Plan) or Protocol Case	8.25	9.00	4.53	4.00	0.15/100 cases	0.2–0.8	0.10
Simple Plan			2.52	4.00			
Intermediate Plan			2.70	4.00		0.05–0.2	
Special Procedures (hr/case)							
TBI	12.17	2.00					
Stereotactic Cranial	4.00	8.00					
Stereotactic Body	‐	10.00					
Electron Total Skin	20.00	‐					
Average Time	12.06	6.67		10.00	0.5/100 cases	0.1–0.5	0.30
Brachytherapy Procedures							
Average Time/Fraction (HDR/LDR)			3.32	4.00	0.20/100 fx		
Interstitial Implants [hr/procedure]	11.84	7.00		10.00	0.50/100 cases	0.2–0.5	0.30
Imaging Procedures (hr/case)							
Multi‐Imaging for Planning	2.50	0.43		3.00	0.15/device		
On‐Line IGART Support (hr/case)	1.00	0.58		3.00	0.15/device		
Equipment Support and QA (hr/yr)	Commission			Maintain			
Multi‐Mode Linac	855.00			400.00	0.20/device	1.0–1.5	0.40
Single Mode Linac	432.50			200.00	0.10/device	0.5–1.0	0.30
IGART CT	131.67			100.00	0.05/device	0.0–0.5	0.10
Treatment Planning System (Server)				200.00	0.10/device	0.5–0.1	0.20
Initial Commissioning [hr/system]	729.29						
Software Upgrades [hr/upgrade]	89.17						
Othovoltage Unit	163.60			100.00	0.05/device		0.10
Radiographic Simulator	95.00			100.00	0.05/device	0.1–0.5	0.10
CT ‐ simulator	173.00			100.00	0.05/device		0.10
4DCT/Gating System	184.00			200.00	0.10/device		0.20
MRI simulator	263.33			200.00	0.10/device		0.20
PET‐CT Scanner	362.00			200.00	0.10/device		0.20
HDR,PDR,LDR units	177.86			200.00	0.10/device	0.1–0.4	0.20

A practical implementation of this approach uses a grid of FTE weights for each procedure type (T) and type of personnel (P).^(^ ^6^ ^)^ An FTE weight describes the completion of a specified standardized number ‘n’ of clinical procedures (e.g., 0.8 FTE physicists per 1,000 treatment plans) or in support of a specified standardized number of radiotherapy pieces of equipment (e.g., 0.6 FTE physicists per linear accelerator). Each FTE weight may be derived from Eq. (1) and justified by the time required per procedure.

(2) FTE Weight (P,n,T)=FTE (P,n,T)

where the standardized number ‘n’ is determined by the time required per procedure. The FTE demand for N procedures *actually completed* in a work year is then obtained by rescaling:

(3) FTE (P,N,T)=FTE Weight (P,n,T)×N/n

Table 2 details FTE(P,n,T) weights for medical physicists using the times per task listed in Table 1 from other jurisdictions, and accounting for progression along the learning curve and process streamlining expected in the future. FTE weights for the other professionals are also listed, and are adjusted according to recent staffing levels and experience. Compared with previous Ontario algorithms, we have added a new entry for case complexity, introduced a new category for information technology specialists, and we have explicitly included FTE loss due to paid holidays and vacation (0.1 FTE per employee). An important feature of our algorithm is that the additional time away for professional development and maintenance of competency (conferences, courses, or site visits) is incorporated in an allocation of 20% for development activities per physicist.

**Table 2:** Spreadsheet showing the Ontario 2011 recommendations of FTE for unit workload specified in the shaded green cells in column 2. For the training and education of specialists, a baseline value is used for the physicist calculation only where indicated.

		*FTE Weighting*					
		*Physics*			*Engineering*		
*Item*	*Workload*	*Physicist*	*Assistant*	*Dosimetrist*	*Electronics*	*Mechanical*	*Computer Support*
Clinical Procedures and Services						
All radiation beam/source therapy ‐ includes external beam therapy and brachytherapy	1	0.0005	0.0002	0.0020	0.0002	0.0001
Complexity bonus increment for inverse IMRT including tomotherapy, clinical trial protocols, gated beams, 4D plans, multi‐modality image fusion (cases/yr).	1	0.0015		0.0030		0.0003
External beam ‐ special procedure bonus increment (total body X or electron, radiosurgery) (cases/yr)	1	0.0050	0.0025	0.0010	0.0010	
Brachytherapy ‐ LDR or HDR (fractions/yr)	1	0.0020	0.0005	0.0004		
Brachytherapy ‐ interstitial seed implants (cases/yr)	1	0.0050	0.0020	0.0020		
Radiotherapy Equipment Support							
Accelerators (all linacs, including tomotherapy and robotic linacs)	1	0.20	0.30		0.30 0.10	
Major ancillary RT equipment: TPS (1 per vendor per 10 workstations), PET‐CT, MR‐Sim, 4D CTsim, HDR	1	0.10	0.05		0.20 0.05	0.10
Minor ancillary RT equipment: X‐ray Sim, CT‐Sim, LDR unit, Gamma Knife, orthovoltage unit, ultrasound unit, gating/motion monitoring device	1	0.05	0.03		0.10 0.05	
Training and Education of specialists							
Radiation Oncology Residents^*^	1	0.11		0.05		
Radiation Therapy Students	1	0.02		0.05		
Clinical Physics Residents^*^	1	0.20		0.05		
Medical Physics Graduate Students^*^	1	0.20				
Administration & Other Duties							
Administrative workload per staff category (Human Resources)	(0.1 * number of physicists) + (0.02 * number of other staff), added to physicist FTE only						
Administration (by Chief, Radiation Safety Officer)	(0.15 ^*^ number of physicists), added to physicist FTE only						
Clinical development, conference attendance, courses, site visits	Total physicist FTE increased to allow 20% effort						
Time away for paid holidays and vacation (FTE per employee)	Total staff FTE increased to allow 10% absence						

* A baseline value of 0.1 is used for the calculation of physicist FTE only.

### B. Method 2: Staffing guidelines “per case”

This method determines staffing numbers by dividing the number of cases treated per year by the number of employees (actual or projected) of a particular type in the same year. The ratio of cases per FTE can theoretically be traced back to the “time per procedure” method (Method 1) by determining all of the clinical or technical aspects associated with completing a treatment case. The value of this ratio then reflects the average time spent on an array of procedures performed (treatment planning, appliance design, dosimetry, quality assurance) in support of a single case. The staffing ratio for personnel (P) would then be calculated as a reciprocal, averaged over a sampling of similar cases (T):

(4) FTE Ratio (P)=Average [1/(Σ FTE(1,T)]

where the resultant FTE ratio is in units of “annual treated cases per FTE”, and FTE(1,T) is the fractional FTE demand per case (using Eqs. 1 or 3).

A sample calculation illustrates the “averaging” link between Methods 1 and 2. Consider a physicist's tasks associated with planning and delivering external beam treatment, including treatment planning, chart checking, dose calculations, accelerator calibration, ongoing consultation, and patient‐specific dosimetry. If these collectively consume a total of approximately 7 hours per typical case (see representative times in Table 1), then the FTE demand per single case would be approximately (7 hrs/1950 hrs)=0.0036 FTE per case, and the cases/FTE ratio would be the reciprocal 1/0.0036=279 cases per FTE.

### C. Supply–demand staffing scenarios

For planning human resources over time, we have developed a “4Rs” model of supply and demand that considers four elements: Requirements (or supply), Retention, Recruitment, and output of Residency training programs (named after the well known 4Rs radiation biology model). Requirements are set by the preceding staffing algorithms. Retention is used to offset attrition caused by departures to opportunities elsewhere, resignations, or retirements. Recruitment refers to attracting out‐of‐province candidates to Ontario. The physics residency program consists of 20 funded residency positions that yield approximately eight provincially‐qualified medical physicists per year (allowing for program length being somewhat variable and some residents not completing the program), with seven graduates typically choosing to remain in the Ontario workplace.

Our staffing levels are modeled using a simple compartment in which supply and departure rates (FTE gains and losses per year) are considered. The net number of FTEs in the workforce at the end of any given year is calculated from:

(5) N_end=N_start+N_recruit_external+N_recruit_residency−N_lost

where:

N_end=number of FTE staff members at the end of a fiscal period,

N_start=number of FTE staff members at the start of a fiscal period,

N_recruit_external=rate of arrival of FTE staff members recruited from outside of Ontario,

N_recruit_residency=rate of hiring FTE residents from the Ontario training program, and

N_lost=rate of departure of FTE staff members lost from Ontario during the fiscal period.

We have developed an Ontario staffing forecast for the period 2010–2020 which is presented in the results section. This is a simplified version of the computer‐aided model described by Mills et al.^(^ ^19^ ^)^

To investigate the use of the Ontario algorithm (Method 1) in other Canadian jurisdictions, the algorithm was coded in a simple spreadsheet (MS‐Excel) and emailed to 37 Canadian radiation treatment programs. In addition to the workload parameters, the survey also asked for the number of physics staff currently employed and the number that the center felt would be appropriate for the workload, to provide a guide to assess the appropriateness of the numbers calculated by our algorithm. During 2010, there were very few open positions in Canada and the data were not corrected for staff vacancies.

## III. RESULTS

**Figure 2:**
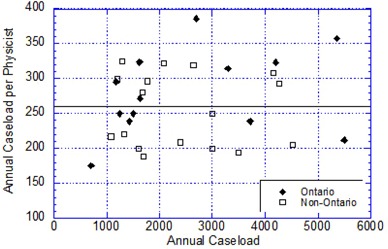
The actual annual caseload per medical physicist at individual treatment centers as a function of annual caseload at that center in September 2010. The overall average number of cases per physicist is 260 (solid line), with values of 280 and 245 for Ontario and the rest of Canada, respectively.

Thirty‐two centers responded to the survey, 13 from Ontario and 19 distributed across Canada from Victoria to Halifax, an 87% response rate. According to these responses, the mean value of treated cases per physicist in Canada in 2010 was 260, with values ranging from 80 to 386. The data show that the caseload per year per physicist is independent of the annual caseload (Fig. 2). The wide scatter of the data is indicative of the challenge associated with using annual caseload alone to determine and compare physicist staffing.

The data from Ontario refer to a year when 33,347 cases were treated and 26,979 cases were designated as newly‐diagnosed treated cases. Despite the standardized reporting practices in Ontario, the Ontario physicist staffing level per case shows considerable variation across centers with a range of 175 to 357, mean of 280. Causes for this variation include differences in the maturity level of each center, caseload volume and complexity, relative number of retreatments during the year, complexity of treatment techniques, daily hours of clinical operation, equipment commissioning load, and new capital construction.

**Figure 3:**
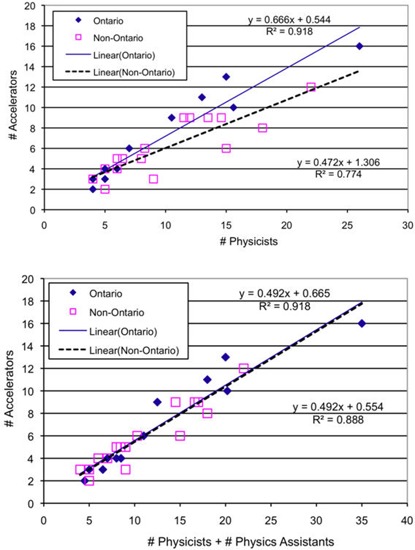
Correlation between the number of megavolt accelerators and the number of physicists (top) or total number of physicists plus physics assistants (bottom).

Interestingly, the ratio of the number of megavolt accelerators to the number of physicists differs between Ontario and the rest of Canada, but is highly correlated with the total number of physicists plus physics assistants (average $R^{2}=0.9$) for Canada as a whole, illustrating the differing levels of use of physics assistants across the country (Fig. 3). It should be noted here that apart from Québec, where there are no plans to hire physics assistants, the only centers not employing physics assistants are smaller remote centers with smaller caseloads.

**Figure 4:**
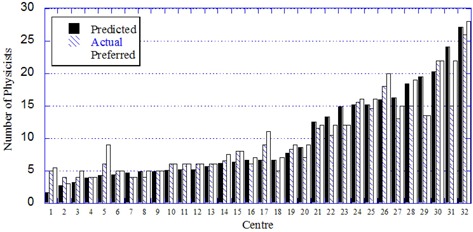
Correlation between the actual, calculated, and preferred numbers of FTE medical physicists in 32 Canadian centers.

The application of the Ontario staffing algorithm for all participating Canadian centers is illustrated in Fig. 4, which plots the differences between the numbers calculated by the workload‐specific algorithm, the current (actual) staffing, and the “desired” numbers expressed by the survey respondents. In general, the staffing algorithm correlates well with the existing situation, with 17/31 (54.8%) of calculations being within ± 1 FTE FTE of the current physicist staffing. One center appears to be severely understaffed for the declared complexity of cases and heavy teaching workload, with an actual staffing of 15 physicists compared to a calculated requirement of 24. Only one center admitted to having more physicists than “desired”, 11 of the 32 centers didn't feel the need for additional physicists, and the remaining 20 centers expressed a need for a few additional staff (Fig. 4).

Using the detailed staffing algorithm together with the data collected from 32 Canadian centers responding to the survey, we have calculated staffing needs for the year 2010 for all staff categories, the average values given in Table 3. We then calculated the average values for the ratio of cases/FTE for Canada as a whole and for Ontario specifically. The two averages reported in Table 3 are not statistically different; however, these numbers illustrate once again the inadvisability of the “one size fits all” scenario. For example, for the physicists, our workload‐specific algorithm calculated a range of values depending on the variation in reported workload among the centers from 163 treated cases per physicist (for a large academic center with a high complexity factor and large number of students/residents) to 418 (for a small center with few complex treatments, no special procedures, and no students/residents). For large‐scale forecasting, the following number of treated cases per FTE per year were determined: 260 for medical physicists, 700 for physics assistants, 300 for medical dosimetrists, 600 for electronics engineering technologists providing in‐house service, 1200 for mechanical engineering technologists, and 2000 for information technology specialists. Coincidentally, the current average staffing of physicists across Canada measured by our survey is also 260 treated cases per year, although the distribution of numbers differs for each center, as can be seen from Fig. 4.

**Table 3:** Summary of staffing prediction in terms of annual caseload per FTE using our algorithm for each staff group and 32 Canadian cancer centers.

				*Engineering*		
	*Physicist*	*Physics Assistant*	*Dosimetrist*	*Electronics*	*Mechanical*	*IT Suppo*
Average (Canada)	263.3	692.7	317.7	626.1	1269.5	2508.0
Standard Deviation	55.0	110.4	52.9	142.1	219.9	776.0
Minimum	163	421	199	277	679	1427
Maximum	418	877	409	915	1665	4812
Average (Ontario)	254.7	694.9	300.1	601.3	1252.0	2143.8
Ontario SD	29.1	72.2	50.5	117.9	163.0	596.0
Minimum	202	582	199	419	994	1452
Maximum	316	818	365	803	1554	3292
Recommended	260	700	300	600	1200	2000

We recommend that the detailed workload‐sensitive algorithm be used to determine staffing for a particular center, whereas a ratio of 260 treated cases per physicist (determined independently through a survey of existing staffing and by applying the workload‐sensitive algorithm on a center‐by‐center basis to 32 Canadian centers) be used for larger scale planning — for example, on a provincial or national basis.

### Staffing Projections for 2010–2020

**Figure 5:**
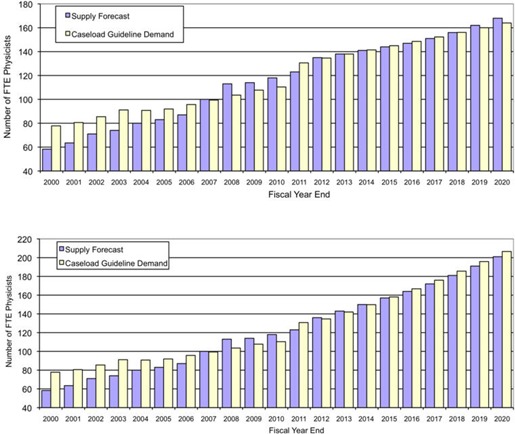
Calculation of physicist staffing levels for Ontario for the period 2000–2020 using our supply model, assuming: (top) 2.5% growth in caseload at the current rate of radiotherapy utilisation, and (bottom) 5.5% growth to improve the utilisation rate from 2011. The radiotherapy demand is calculated using 300 cases per physicist until 2010 and the updated guideline of 260 cases per physicist from 2011.

The recommended value for medical physicists (260 annual cases per FTE) was then used to forecast provincial needs for the period 2010 to 2020, reported in Fig. 5. The supply–demand scenario was based on the following assumptions:

1. Cancer incidence of new cases increases by 2.5% per year from a baseline of actual new cases in Ontario for 2009, reduced from the 3.0% assumed for the previous decade.
2. The projected staffing levels allow for coverage of 10 treatment hours per day using adjustments of an individual employee's starting time but excluding overtime hours. If the work day is extended beyond 10 hours, additional staffing may be needed to cover early morning and late evening shifts, as well as the repair, maintenance, and quality assurance of treatment equipment. Extended hours of operation also have implications for the more frequent replacement of major equipment (e.g., linear accelerators, and associated commissioning workload).
3. The starting number of physicists is the actual number currently in the province (123 FTE) in the Fall of the year 2010.
4. The attrition rate is set at seven physicists per year for the period 2010–2015 to include departures and retirements. After 2015, the rate is reduced to five physicists per year based on the workforce age distribution.
5. The recruitment from out‐of‐province is initially set at five physicists per year, but tapers down to nil by 2020.
6. The recruitment from the Ontario residency training program is fixed at seven physicists per year based on historical patterns and assuming ongoing government funding.^(^ ^37^ ^)^
7. The clinical practice does not change over the time period (i.e., there are no changes in the algorithm parameters such as the percentage complexity).

The upper plot does not include increases in radiotherapy utilization. The utilization rate of radiotherapy in Ontario has been measured^(^ ^25^ ^,^ ^41^ ^)^ and found to be relatively low because of poor access to services and patient referrals in some regions of Ontario. There is now a concerted plan by Cancer Care Ontario to enhance the utilization rate over the next decade, and our staffing model accounts for this government‐driven initiative and the expansion of accessible services. The utilization rate is forecasted to rise from 35.5% to 48% of incident cancer cases by 2020 and the second plot in Fig. 5 includes a growth rate in cases per annum of 5.5% per annum to reflect this anticipated increase.

## IV. DISCUSSION

The impact of implementing new technologies and procedures in Ontario may be inferred from the change in annual treated cases per FTE physicist from 400 in the pre‐IMRT era to approximately 280 in the post‐IMRT era. This represents a 30% change in workload due to the progressive implementation of advanced technology and the support of new treatment techniques. In the year 2000, very few patients were treated with inverse‐planned IMRT in Ontario; at present IMRT accounts for approximately 25% of the radiotherapy cases and this rate shows every indication of increasing and is consistent with the provincial cancer plan.^(^ ^31^ ^)^

This experience echoes the US trend where the median number of annual new patients per FTE for all the cancer centers surveyed by the American College of Radiology (ACR) has declined from 424 (pre‐IMRT era) to 304 (IMRT era), also a 30% decrease.^(^ ^26^ ^)^ A direct comparison of the absolute caseloads in the US and in Canada is difficult because of different workload measures used; there is also a different mixture of comprehensive cancer centers and stand‐alone centers.^(^ ^42^ ^)^ The question of whether the case per FTE will bottom‐out or rebound in the next decade through streamlining of IMRT and IGRT procedures is still being debated.^(^ ^43^ ^)^

The American College of Radiation Oncology (ACRO) Red Book proposes a standard of 300–400 annual new patients per FTE physicist, assuming a 25% IMRT load.^(^ ^44^ ^)^ The total number of treated cases (including retreatments) would actually be up to 22% higher based on the Ontario experience. This would result in an excessive workload of 375–500 cases per FTE physicist, illustrating the need for absolutely clear definitions of caseload parameters. The American College of Radiology (ACR) reports an average ratio of 196 “new patients per physicist” for comprehensive cancer centers, and 220 for hospital‐based centers with admissions exceeding 600 patients per year.^(^ ^45^ ^)^ If we apply the above‐mentioned 22% correction for retreatments, the ACR ratios then cover a range of 240–270 patients per year per physicist, in agreement with our algorithm results for Ontario (257) and Canada (259). The ACR cautions that “each facility should, when comparing to … national averages, consider the patient population, range and complexity of services provided, and any staff duties outside of core duties…”. Klein has also emphasized that the application of the national ACR values (from the year 2008) would have led to serious understaffing in a specific cancer center; local variables must be taken into account.^(^ ^20^ ^)^ Also of note, ACR values are being updated over a number of years, during which time advanced technology (e.g., IMRT) is gradually being implemented; therefore, a running average may not be a suitable basis for recommendations.

Until recently, radiation therapy resources in Canada have been located mainly in large urban centers. The desire to offer patients treatment in their community, leading to a decentralization of radiotherapy facilities over the last 10 years and the construction of smaller facilities either remote from, or on the edges of, large urban centers, is another factor contributing to the recent increase in physics staffing. The plot in Fig. 3 shows that a typical small Canadian center with three treatment machines currently has a minimum of four medical physicists, independent of the caseload. This situation may change with the introduction of newer satellite centers relatively close to a larger center and resourced from that larger center without requiring extensive travel.

Our algorithm is novel in that it also explicitly accounts for professional development activities, adding an increase of 25% per physicist for such factors as clinical development, continuing education, and attendance at conferences.

Our work predicts an increase in requirement of physicist staffing on the order of 33% over the next 10 years, requiring a recruitment of 16 physicists per year for Ontario, taking into account predicted retirements and historical attrition. This is higher than the 20% increase over current values predicted by the US Center for Health Workforce Studies,^(^ ^27^ ^)^ which estimates a required recruitment of board‐eligible radiation oncology physicists of more than 190 per year for the US.

## V. CONCLUSIONS

In summary, we have analyzed a decade of experience with radiotherapy in the province of Ontario, Canada, where data collection is standardized for a large cancer treatment system. The rapid implementation of new technology has escalated the workload per case considerably, with a heightened awareness for patient‐specific quality assurance and safety. Accordingly, we have updated our staffing algorithm based on a grid of FTE coefficients for each type of staff functioning as a team providing medical physics services in a radiation treatment program. The algorithm was tested with data from 32 centers across Canada and proved to be sensitive to local situations including clinical services, academic and training activities, and administration. Using the calculated FTE complement for a wide range of staff and cancer centers, we determined staffing ratios (treated cases per FTE) that may be useful for large‐scale planning at provincial or national levels. In conclusion, we recommend the use of a detailed workload‐specific algorithm for local staff planning, while simple FTE caseload ratios may be used for larger‐scale planning. The ultimate goal of any staffing plan is to offer cancer patients state‐of‐the‐art radiotherapy in a timely and safe manner.

## ACKNOWLEDGMENTS

The authors wish to thank Drs. Brendan McClean and Alan Hounsell for providing the staffing reports produced by IPEM. We thank our colleagues from across Canada for taking the time to supply valuable data and feedback.
